# “Green” Extraction and On-Site Rapid Detection of Aflatoxin B1, Zearalenone and Deoxynivalenol in Corn, Rice and Peanut

**DOI:** 10.3390/molecules28073260

**Published:** 2023-04-06

**Authors:** Zijing Li, Zepeng Li, Xintong Li, Qi Fan, Yinuo Chen, Guoqing Shi

**Affiliations:** School of Chemistry and Biological Engineering, University of Science and Technology Beijing, Beijing 100083, China; lizijing09@163.com (Z.L.); 13021230300@163.com (Z.L.); m202110884@xs.ustb.edu.cn (X.L.); qi.fan17@outlook.com (Q.F.); 18612203588@163.com (Y.C.)

**Keywords:** lateral flow immunoassay, aflatoxin B1, zearalenone, deoxynivalenol, rapid detection

## Abstract

The common mycotoxins in polluted grains are aflatoxin B1(AFB1), zearalenone (ZEN) and deoxynivalenol (DON). Because of the potential threat to humans and animals, it is necessary to detect mycotoxin contaminants rapidly. At present, later flow immunoassay (LFIA) is one of the most frequently used methods for rapid analysis. However, multistep sample pretreatment processes and organic solvents are also required to extract mycotoxins from grains. In this study, we developed a one-step and “green” sample pretreatment method without using organic solvents. By combining with LFIA test strips and a handheld detection device, an on-site method for the rapid detection of AFB1, ZEN and DON was developed. The LODs for AFB1, ZEN and DON in corn are 0.90 μg/kg, 7.11 μg/kg and 10.6 μg/kg, respectively, and the working ranges are from 1.25 μg/kg to 40 μg/kg, 20 μg/kg to 2000 μg/kg and 35 μg/kg to 1500 μg/kg, respectively. This method has been successfully applied to the detection of AFB1, ZEN and DON in corn, rice and peanut, with recoveries of 89 ± 3%–106 ± 3%, 86 ± 2%–108 ± 7% and 90 ± 2%–106 ± 10%, respectively. The detection results for the AFB1, ZEN and DON residues in certified reference materials by this method were in good agreement with their certificate values.

## 1. Introduction

Mycotoxins are among the secondary metabolites released by moulds, particularly fungi, which mainly include aflatoxin B1 (AFB1), zearalenone (ZEN) and deoxynivalenol (DON) [1,2,3,4]. The International Agency of Research on Cancer (IARC) classified AFB1 into Group 1, which includes substances with sufficient evidence to support their carcinogenicity in humans [5,6,7,8,9]. Structurally, zearalenone is similar to 17β-oestradiol, which can cause abortion, stillbirth and teratogenesis, and can cause symptoms of central nervous system poisoning and even death [2,10,11]. Deoxynivalenol (DON), also known as vomiting toxin, can result in vomiting [2,12,13]. Mycotoxins may be produced during the production, processing, transport and storage of grain [14,15,16,17]. According to FAO data [18], approximately 25% of wheat, corn, sorghum and rice are polluted by mycotoxins every year. In addition, studies have shown that most mycotoxins cannot be eliminated in the food processing and cooking process [19]. Therefore, some risks of mycotoxin exposure cannot be ignored in food and its products. If rapid and on-site methods can be developed to detect mycotoxin contamination in food in the fields, factories, grain depots, shopping malls and even in homes, people can find dangerous foods more efficiently, thereby reducing health risks to humans and animals.

At present, mycotoxin detection methods in grain can be mainly divided into two categories. One is quantitative analytical methods, including high-performance liquid chromatography (HPLC) [17], liquid chromatography-mass spectrometry (LC-MS) [20,21] and gas chromatography-mass spectrometry (GC-MS) [20,22], which use large-scale instruments with high sensitivity and accuracy for the quantification of mycotoxins. However, the sample pretreatment process of those methods is normally laborious and requires a professional person to operate. The other is rapid analysis methods based on immunological analysis methods, such as enzyme-linked immunosorbent assay (ELISA) [23], fluorescence immunoassay (FFIA) [24], and lateral flow immunochromatography (LFIA) [25,26,27,28], but they also need multistep sample pretreatment processes to prepare test solutions. For example, Li et al. developed a quantum dots (QDs) fluorescence LFIA method to simultaneously detect AFB1, ZEN and DON, in which the sample was extracted by methanol: water (60:40, V/V). After vortexing and centrifugation, the supernatant was diluted with PB (0.01 M, pH 7.4) at a ratio of 1:6 to prepare the solution to be tested [29]. Similar sample pretreatment strategies have been used in other studies that use immunoassays to detect mycotoxins [27,30,31,32,33]. Not only is this process time-consuming and laborious, but the use of organic solvents increases the risk of endangering human health and polluting the environment. In addition, users are also faced with an increase in storage, transport, management and other costs because of the use of organic solvents, such as methanol and ethanol, which are inflammable and explosive dangerous goods. Obviously, the methods mentioned above are not suitable for on-site and household use, so it is necessary to find a “green” and environmentally friendly solution to extract mycotoxins from foods and develop a simple sample pretreatment method that does not rely on bulky instruments.

The water solubilities of AFB1, ZEN and DON are very different; DON is easily soluble in water, AFB1 is hardly soluble in water, and ZEN is almost insoluble in water. Compared with DON, AFB1 and ZEN are difficult to extract with aqueous solutions, which may be the reason why organic solvents are often used for extraction in existing methods. In our previous work, we found that fatty alcohol polyoxyethylene ether (AEO) surfactants can increase the partition coefficient of ZEN in the water phase of an oil-water mixture system [34]. AEO surfactants are non-ionic surfactants that are often used as detergents, defoamers, emulsifiers, levelling agents, etc. AEO surfactants have low skin irritation and good biodegradability, so they are friendly to the human body and environment compared to organic solvents. Based on this, we were inspired to attempt to extract ZEN, AFB1 and DON from grain with solutions containing AEO. If ZEN can be extracted, AFB1 and DON with lower relative lipophilicities will also be extracted. Therefore, we can establish a sample pretreatment method without using organic solvents. By combining this method with LFIA test strips and a hand-held detection device, a method for the rapid detection of AFB1, ZEN and DON, three mycotoxins in grain, can be established, which will be suitable for field and even family use.

In this study, we first studied the extraction efficiency of AEOs with different chemical structures for AFB1, ZEN and DON in grain samples. Then, we selected a specific AEO solution that can simultaneously extract these three mycotoxins, and optimized the concentration, volume and extraction time of the extractant solution. Finally, we established a LFIA method that can be used for on-site rapid detection of AFB1, ZEN and DON.

## 2. Results and Discussion

### 2.1. Optimization of the Sample Preparation

#### 2.1.1. Optimization of the Composition of the Mycotoxin Extraction Solution

**Figure 1:**
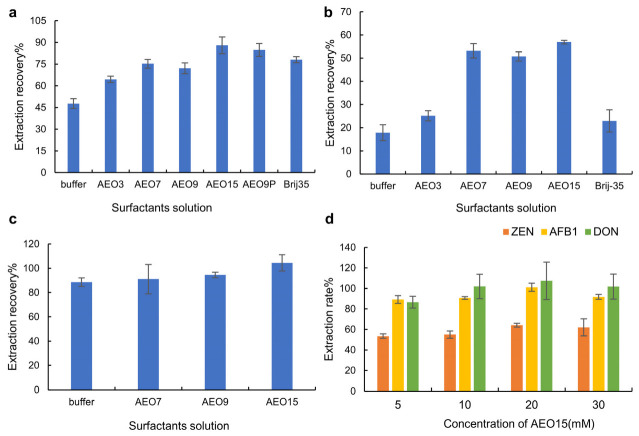
The influence of surfactants on the extraction rate of mycotoxins in corn: (**a**) AFB1; (**b**) ZEN; (**c**) DON. (**d**) The influence of different concentrations of AEO15 on the extraction rates of AFB1, ZEN and DON.

In this study, it was necessary to first screen mycotoxin extraction solutions without organic solvents that are suitable for extracting AFB1, ZEN and DON from grain, to increase the convenience of detection and achieve the goal of “green” extraction. Certified reference materials (CRMs) of blank corn flour and of corn flour containing AFB1, ZEN and DON respectively were used as grain samples to screen the surfactants in the extraction solution. The surfactant solution with the highest extraction rate was selected as the mycotoxin extraction solution. We first investigated the extraction effect of AEO surfactants on AFB1 in CRMs of corn flour containing AFB1. As shown in Figure 1a, compared with the buffer, the extraction rates of the AEO7, AEO9, AEO15 and Brij35 solutions were higher than 70%; in particular, the extraction rate of the extraction containing AEO15 was the highest, reaching 87.96%, indicating that these extraction solutions could effectively extract AFB1 from corn flour. Next, we investigated the extraction efficiency of AEO surfactants on ZEN in CRMs of corn flour containing ZEN. The extraction rates of solutions containing surfactants AEO7, AEO9 and AEO15 for ZEN in corn flour were significantly higher than those of AEO3 and Brij-35, as shown in Figure 1b. Therefore, we next investigated the extraction rate of DON in CRMs of corn flour containing DON, because of the high extraction efficiency of the extraction solutions containing AEO7, AEO9 and AEO15, respectively. As shown in Figure 1c, there was no significant difference in the extraction rate of DON from corn flour by the extraction solutions containing AEO7, AEO9 and AEO15. Considering the influence of the extraction rate and matrix effect on the extraction of AFB1, ZEN and DON from grain, the extraction solution containing AEO15 was selected as the optimal extraction solution for the extraction of the three mycotoxins from corn flour. Compared to extractants containing organic solvents [35,36,37], the AEO15 solution has a similar extraction efficiency for AFB1, ZEN and DON, but is more “green”.

#### 2.1.2. Optimization of the Concentration of Mycotoxin Extraction Solution

Then, the concentration of the AEO15 solution was optimized for the extraction of AFB1, ZEN and DON from corn flour. First, the effect of different AEO15 concentrations on the extraction rate of AFB1 in corn flour was investigated. As shown in Figure 1d, the extraction rates of the four groups were between 80% and 110%, which met the detection requirements. Next, the effect of the AEO15 concentration on the extraction rate of ZEN in corn flour was investigated. As shown in Figure 1d, when the concentration of AEO15 was 10 mM, the extraction rate was 54.95%. Increasing the AEO15 concentration did not significantly increase the extraction rate. Finally, the effect of the AEO15 concentration on the extraction rate of DON from corn flour was investigated. The results in Figure 1d showed that the extraction rates from the investigated AEO15 solutions were all between 80% and 110%, and there was no significant difference among the groups. Because the extraction solution containing 20 mM AEO15 had the highest extraction rate for AFB1, ZEN and DON in corn flour, 20 mM was selected as the optimal concentration of AEO15 in the extraction solution.

#### 2.1.3. Optimization of Extraction Time

**Figure 2:**
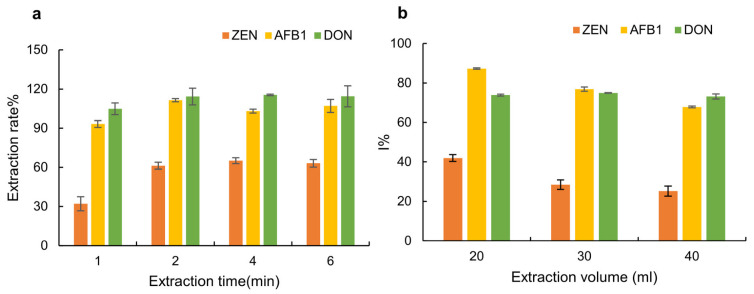
Influence on the I% of AFB1, ZEN and DON of (**a**) different extraction times of 1, 2, 4 and 6 min; and (**b**) different extraction volumes of 20, 30 and 40 mL.

The effects of extraction time on the extraction rates of AFB1, ZEN and DON in grain samples were also investigated. To investigate the effect of extraction time on the extraction rate, the selected extraction solution and sample (20 μg/kg) were mixed at a volume ratio of 1:30, and 1, 2, 4 and 6 min were set as the extraction times. After standing for 5 min, the influence of different extraction times on the extraction rate was investigated. As shown in Figure 2a, the extraction rate was close to 100% for AFB1 and DON after 1 min of extraction, but for ZEN, the extraction rate after 2 min was significantly higher than that after 1 min, and there was no significant difference in the extraction rate at extraction times of 2 min, 4 min and 6 min. Therefore, 2 min was finally selected as the optimal extraction time for simultaneously extracting AFB1, ZEN and DON from grain. Due to the usual steps of vortexing, centrifugation and dilution, the traditional sample pretreatment process requires more than 25 min [30,35], while only 4 min was required in this method, which greatly reduces the time for the entire detection.

### 2.2. Optimization of Preparation Conditions for TRF-LFIA Test Strips

The preparation conditions of the TRF-LFIA test strips for the simultaneous detection of AFB1, ZEN and DON were optimized, as shown in Supplementary Materials: Figure S1. Finally, 0.3 mg/mL AFB1-BSA, 0.05 mg/mL ZEN-BSA and 0.5 mg/mL DON-BSA were selected as coating antigens for the TRF-lateral flow strip. Under the optimized preparation conditions, the detection ranges for AFB1, ZEN and DON in solution were 0.03–0.9 μg/L, 0.3–25 μg/L and 1.0–50 μg/L, respectively.

### 2.3. Optimization of Extraction Volume

One gram of CRMs of corn flour spiked with AFB1, ZEN and DON at concentrations of 20 μg/kg, 60 μg/kg and 1000 μg/kg, respectively, and CRMs of blank corn flour were extracted with 20, 30 and 40 mL of extraction solution under the optimized conditions. The experimental results (Figure 2b) showed that the I% values for AFB1, ZEN and DON (76.9%, 28.5% and 72.2%) were in the detection ranges of AFB1, ZEN and DON, respectively, when 30 mL of extraction solution was used. Therefore, 30 mL was selected as the optimized extraction volume. Compared to the pretreatment processes of existing approaches [35,36,38,39], which use 20 mL methanol or acetonitrile and shaking for 30 min to extract mycotoxins from grains, followed by centrifuging for 15 min and then dilution of the supernatant at different ratios [37], the sample pretreatment method of this work was simple and easy to operate.

### 2.4. Standard Curves

**Figure 3:**
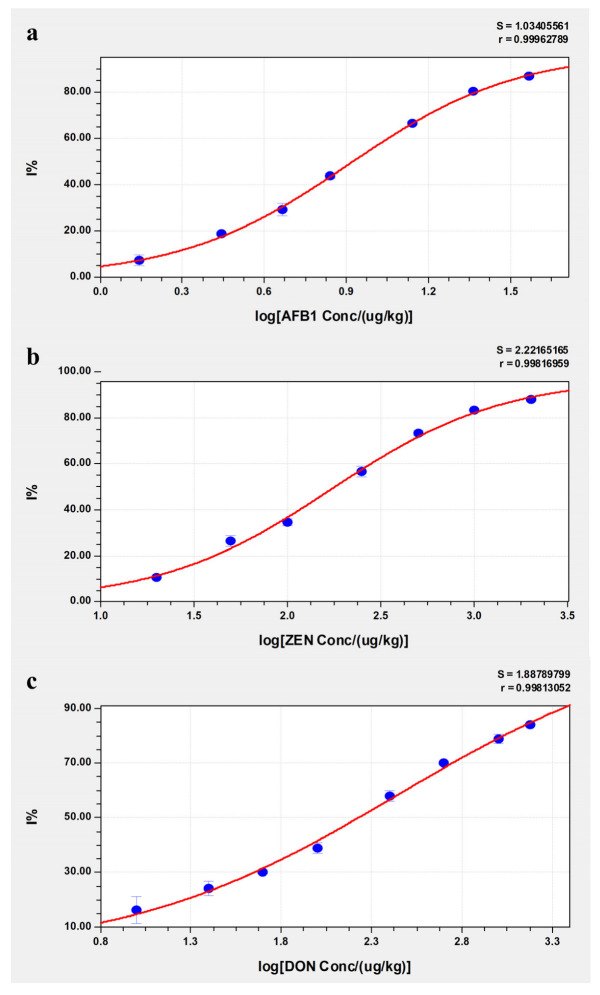
Standard curves for AFB1, ZEN and DON in corn. (**a**) AFB1; (**b**) ZEN; (**c**) DON.

The standard curves for detecting AFB1, ZEN and DON in corn flour are shown in Figure 3. The standard curves of AFB1, ZEN and DON were based on the four-parameter logistic equation, and the working ranges were from 1.51 μg/kg to 40 μg/kg, 20 μg/kg to 2000 μg/kg and 35 μg/kg to 1500 μg/kg, respectively. The LODs of AFB1, ZEN and DON were 0.90 μg/kg, 7.11 μg/kg and 10.6 μg/kg, respectively. The LOQs of AFB1, ZEN and DON were 1.51 μg/kg, 20.3 μg/kg and 35.4 μg/kg, respectively. The maximum residue limits were 20 μg/kg for AFB1, 60 μg/kg for zearalenone and 1000 μg/kg for deoxynivalenol in cereals and their processed products [35], all within the working range of the three standard curves of AFB1, ZEN and DON in this study. As the residue limits of AFB1, ZEN and DON are quite different, the extracts must be diluted for detection at different ratios for different mycotoxins in other studies [36,40]. However, the method developed in this study can simultaneously extract three mycotoxins directly from grains without dilution for detection. Therefore, this approach simplifies the operation process and is easier for nonprofessional users, which can help realize on-site testing and home testing.

### 2.5. Recovery of AFB1, ZEN and DON in Grains

To evaluate the accuracy of this method, CRMs of blank corn flour, rice flour and peanut purchased from TMRM were spiked with various concentrations of AFB1, ZEN and DON and detected using the strips.

The recovery was calculated using the following equation:

Recovery% = (C1 − C0)/C × 100%,(1)

where C1 is the detected mycotoxin concentration of the sample, C0 is the background mycotoxin concentration of the sample and C is the spiked mycotoxin concentration. As shown in Table 1, the average recoveries in the three substrates varied from 89 ± 3% to 106 ± 3% for AFB1, 86 ± 2% to 108 ± 7% for ZEN and 90 ± 2% to 106 ± 10% for DON. These results indicated that AFB1, ZEN and DON in corn, rice and peanut can be quantitatively determined simultaneously using the developed method with acceptable precision.

### 2.6. Cross-Reactivity

To test the specificity of the detection method, the standard curves and IC50 values of AFB1 analogues, ZEN analogues and other common mycotoxins in corn were obtained by the same procedure as outlined above, and the cross-reactivity of the different AFB1 analogues, ZEN analogues and other common mycotoxins, including AFB2, AFG1, AFG2, α-ZEL, β-ZEL, α-ZOL and β-ZOL, was calculated using the following equation:

CR% = (IC50 for target mycotoxins/IC50 for the analogue) × 100%.(2)

The test strips for AFB1, ZEN and DON detection had low cross-reactivity with these analogues, which demonstrated that they were highly specific for AFB1, ZEN and DON detection in grains (Table 2). Compared with studies that simultaneously detect multiple mycotoxins [36], the CRs of anti-AFB1 mAbs for AFB2, AFG1 and AFG2 in this method are similar, but the CRs of anti-ZEN mAbs for α-ZOL and β-ZOL are lower than in the previous literature [38]. From the results, we observed that the antibodies used in this study were specific. In applications of multi-mycotoxin determination, this method is valuable to some extent for screening rice and corn samples that contain AFB1, ZEN DON and other mycotoxin analogues.

### 2.7. Assessment of the Trueness

To further verify the reliability of this method, the bias between the results of the established method and the certified concentration (as the reference value) was compared by using the method established in this study to detect the commercial CRMs in Table 3. All CRMs used in this study were from naturally polluted grain samples, and the content of mycotoxins in all materials was confirmed by the Chinese national standard method.

The calculation is as follows:

b% = (X − X_0_)/X_0_ × 100, (3)

where X is the detected mycotoxin concentration of the sample and X_0_ is the concentration of the reference value. Although these materials are derived from natural samples contaminated with mycotoxins, they should still be referred to as CRMs rather than real samples. Here we evaluated the trueness of the method by calculating the bias between the results of the established method and the reference concentrations of the CRMs according to the Eurochem Guide [41]. For the seven CRMs with varying AFB1 concentrations from low to high (maximum residue limits), the bias between the test results of this method and the certified concentration ranged from −17% to 8%, showing that the developed method can quantitatively detect all concentrations below the maximum residue limit concentration. For the six CRMs with different concentrations of ZEN from 32 μg/kg to 750 μg/kg in corn and rice, the bias ranged from −11% to 7%. The bias for the five CRMs containing DON at concentrations from 125 μg/kg to 1310 μg/kg was between −15% and −6%. The results indicate that the trueness, excellent anti-interference ability and accuracy of the method established in this study can meet the needs of quantitative detection of AFB1, ZEN and DON in grains.

## 3. Materials and Methods

### 3.1. Chemicals and Reagents

Time-resolved fluorescent microspheres (TRF-MS, mean diameter: 0.07 μm, solids content: 0.53%) modified with carboxyl groups were purchased from Shanghai Suyuan Biotechnology Co., Ltd. (Shanghai, China), with the best measurement at an excitation wavelength of 365 nm and an emission wavelength of 615 nm. Bovine serum albumin was purchased from Sphere-MFCIS (Nanjing, China). Morpholine ethanesulfonic acid (MES) was purchased from Macklin (Beijing, China). Ethanolamine and 1-ethyl-3-(3-dimethylaminopropyl) carbodiimide (EDC) were purchased from Aladdin (Shanghai, China). N-Hydroxysulfosuccinimide (sulfo-NHS) was purchased from Meryer (Shanghai, China). AFB1-BSA and monoclonal antibodies against AFB1 were produced in our laboratory. ZEN-BSA and monoclonal antibodies against ZEN were purchased from Ucando (Guangzhou, China). DON-BSA and monoclonal antibodies against DON were purchased from Lando Biotechnology Co., Ltd. (Shenzhen, China). AFB1, AFB2, AFG1 and AFG2 standards were purchased from J&K Scientific (Beijing, China). ZEN, α-ZEL, β-ZEL, α-ZOL, β-ZOL, DON and OTA standards were purchased from Yuanye Biotechnology Co., Ltd. (Shanghai, China). CRMs of blank corn flour, blank rice flour and corn flour containing 28 μg/kg AFB1, and 1310 μg/kg, 400 μg/kg and 125 μg/kg DON were provided by TMRM (Beijing, China). CRMs of rice flour containing 14.7 μg/kg AFB1 and 8.3 μg/kg AFB1 were provided by Puxi (Beijing, China). CRMs of corn flour containing 2.2 μg/kg AFB1 and rice flour containing 2.6 μg/kg AFB1 were provided by Wanjia (Henan, China). CRMs of corn flour containing 7.4 μg/kg and 20 μg/kg AFB1, 85 μg/kg and 750 μg/kg ZEN as well as rice flour containing 32 μg/kg, 55 μg/kg, 380 μg/kg ZEN and 536 μg/kg, 720 μg/kg DON were provided by Meizheng (Beijing, China). Nitrocellulose (NC) membranes were provided by Pall Biotech (Maharashtra, India). Glass fibre membranes and absorbent pads were obtained from Kinbio (Shanghai, China). The AEO series of surfactants were purchased from Usolf (Shandong, China).

### 3.2. Apparatus

The XYZ HM3010 dispensing platform was purchased from Jiening Biotechnology Co., Ltd. (Shanghai, China). A guillotine cutter was purchased from the ANTOKUN Co., Ltd. (Hangzhou, China). A YSF-1000 158 Dry Fluorescence Immunoassay Analyser was obtained from Perfemed (Beijing, China) for reading strips.

### 3.3. Preparation of mAb-TRF Nanoparticle Conjugates

First, 100 μL TRF-MS (0.5%) was added to a centrifuge tube. After 15 min of sonication, 200 μL of EDC (0.25 mg/mL) and sulfo-NHS (2 mg/mL) in MES buffer (50 mM, pH 5) were added, and the solution was shaken at room temperature for 20 min. It was then dialyzed overnight in PBS buffer (10 mM, pH 7.2) to remove EDC and sulfo-NHS. After dialysis, certain amounts of AFB1 antibodies (0.15 mg/mL), ZEN antibodies (0.15 mg/mL), DON antibodies (0.40 mg/mL) and chicken IgY antibodies (0.30 mg/mL) were added to the solution and kept at room temperature for 1 h 30 min. Then, the microspheres were blocked with BSA (1 mg/mL) for 30 min. Finally, the preservation solution for storage (PB buffer, 5 mM, pH 7.2, containing 5% sucrose, 0.25% Tween 20 and 0.1% Proclin 300) was added to the conjugates and stored in a refrigerator at 4 °C.

### 3.4. Preparation of the TRF LIFA Test Strip

**Figure 4:**
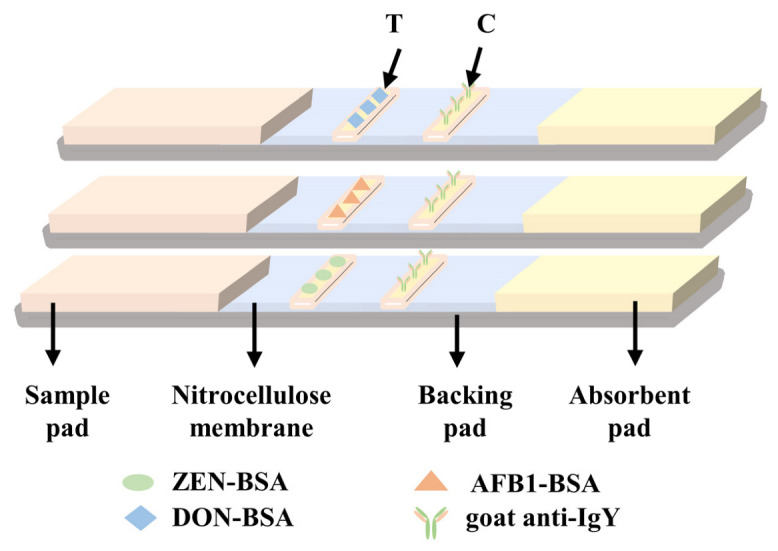
Schematic representation of the immunochromatographic test strip for AFB1, ZEN and DON.

As shown in Figure 4, the time-resolved fluorescence immunoassay test strip was constructed with four parts as follows: PVC plastic card, sample pad, NC membrane and absorbent pad. The NC membrane was pretreated for 1 h at 25 °C and a humidity of 55%. The XYZ HM3010 dispensing platform was used to dispense ZEN-BSA, AFB1-BSA and DON-BSA on the T line and goat anti-chicken IgY antibody on the C line of the NC membrane, at a speed of 0.5 μL cm^−1^. The NC membrane was then dried at 37 °C for 12 h. The sample pad, NC membrane and absorbent pad were pasted onto a PVC plastic card. The card was cut into strips and packed into a customized plastic cartridge.

### 3.5. Detection Procedure

The ground grain sample was mixed with the mycotoxin extract in a certain ratio, shaken for 2 min, and allowed to stand for 2 min. Then, 100 μL of supernatant was dropped into the sample hole of the test strip. The fluorescence values of the T lines and C line were read with a hand-held time-resolved fluorescence measuring instrument after 20 min. Then, the concentrations of AFB1, ZEN and DON were calculated by the inhibition rate % and the working curve. The inhibition rate (I%) was calculated using Equation (4):

I% = (1 − (T/C of sample)/(T/C of blank)) × 100%.(4)

### 3.6. Selection of the Mycotoxin Extraction Solutions

The six kinds of AEO surfactants (AEO3, AEO7, AEO9, AEO15, AEO9P and Brij-35) were prepared with PB buffer (pH 7.2, 50 mM, with 0.3% Tween 20) at a concentration of 5 mM as the mycotoxin extraction solution. They were mixed with CRMs of blank corn flour to make the blank extraction solutions. The supernatants of the blank extraction solutions were used to prepare the different concentrations of AFB1, ZEN and DON solutions for the standard curves. Then, the surfactant solutions were mixed with certified corn flour reference materials containing 20 μg/kg AFB1, 85 μg/kg ZEN and 1310 μg/kg DON at a volume ratio of 30:1 (surfactant solution: grain) and shaken by hand for 2 min. After standing for 2 min, 100 μL of the supernatant was dropped onto the sample pad of the test strip for three tests, and the prepared standard curve solutions were dropped at the same time. After 20 min, the test strips were placed in a hand-held strip reader, and the T/C of the T lines and C line were measured. The inhibition rate (I%) of each mycotoxin extract was calculated and compared with the standard curves to determine the actual detection concentration. The extraction rate was calculated using Equation (5):

Extraction rate % = (actual concentration × dilution ratio)/certified reference material concentration × 100%.(5)

The surfactant solution with the highest extraction rate % was selected as the mycotoxin extraction solution. Finally, an optimal surfactant extract with a good extraction effect for the three mycotoxins in grain could be selected.

### 3.7. Standard Curves

One gram of the CRMs of blank corn flour was spiked with 200 μL of AFB1 standard, ZEN standard and DON standard at different concentrations to make spiked samples (AFB1: 0, 0.5, 1.5, 3, 5, 7.5, 15 and 25 μg/kg; ZEN: 0, 25, 50, 100, 250, 500, 1000 and 2000 μg/kg; DON: 0, 25, 50, 100, 250, 500 and 1000 μg/kg). After stirring evenly, the sample powder was placed at room temperature to dry, and the spiked samples were extracted according to the above sample pretreatment steps. Taking the AFB1, ZEN and DON concentrations as the abscissa and the average value of I% corresponding to the spiked samples at each concentration as the ordinate, a standard curve was obtained with Curve Expert 1.4.0 software (https://www.curveexpert.net accessed on 15 May 2022).

### 3.8. LOD and Working Range

According to “The Fitness for Purpose of Analytical Methods” [41], 10 low-concentration samples near the blank value were used to calculate the LOD value. Three times the standard deviation (so′) of 10 samples was used as the LOD value. The LOQ value was used as the lower limit of the working range, and the concentration producing obvious signal abnormalities was used as the upper limit of the working range. LOQ is the analyte concentration corresponding to so′ multiplied by the coefficient ko, where ko = 10. The value of so′ was used as the standard deviation after correction, so′ = so/ √n, where n is the number of samples that were measured.

## 4. Conclusions

In this study, AEO15 was selected from six different AEO surfactants to simultaneously extract AFB1, ZEN and DON from grains. The optimized concentration of AEO15 solution was 20 mM, and the volume of extractant was 30 mL/g grain. It only took 4 min to complete the sample pretreatment process. The LFIA method has been established for the detection of AFB1, ZEN and DON in cereals. The detection ranges for AFB1, ZEN and DON were 1.25–40 μg/kg, 20–2000 μg/kg and 35–1500 μg/kg, respectively. The accuracy of this method was verified by good recovery for spiked samples (from 86 ± 2% to 108 ± 7%) and low biases relative to the reference values of CRMs. Because a nonorganic solvent extraction solution was used and the extracted solution could be tested directly without filtration, separation and dilution processes, the pretreatment process is “green”, simple and rapid. The method established in this study is suitable for field and family use for the detection of AFB1, ZEN and DON in grain samples.

## Figures and Tables

**Table 1:** Recovery of AFB1, ZEN and DON in different grains.

Target Analyte	Grain Sample	Spiked (μg/kg)	Detected (μg/kg)	Recovery (%)
AFB1	Corn	0	ND ^1^	ND
		7.5	6.71	89 ± 3
		20	21.3	106 ± 3
	Rice	1.5	1.42	94 ± 8
		15	13.9	93 ± 3
		40	41.2	103 ± 9
	Peanut	1.5	1.39	93 ± 10
		7.5	7.27	97 ± 4
		40	36.5	91 ± 2
DON	Corn	20	20.6	103 ± 8
		200	200	100 ± 8
		1000	902	90 ± 2
	Rice	50	51.7	103 ± 6
		200	201	101 ± 2
		1000	913	91 ± 6
	Peanut	50	53.1	106 ± 10
		200	174	87 ± 4
		1000	956	96 ± 1
ZEN	Corn	25	24.2	97 ± 11
		200	196	98 ± 2
		750	688	92 ± 5
	Rice	25	26.3	105 ± 3
		200	174	86 ± 2
		750	742	98 ± 7
	Peanut	25	27	108 ± 7
		200	191	96 ± 5
		750	786	105 ± 4

^1^ Not detected.

**Table 2:** Cross-reactivity between AFB1, ZEN, DON and their analogues.

Target Analyte	IC50 (μg/kg)	Analogue	IC50 (μg/kg)	CRs (%)
AFB1	6.54	AFB2	105	6.22
		AFG1	76.9	8.50
		AFG2	358	1.83
		ZEN	>2000	<0.33
		DON	>2000	<0.33
ZEN	183	α-ZEL	>2000	<9.15
		β-ZEL	857	21.4
		α-ZOL	>2000	<9.15
		β-ZOL	1349	13.6
		AFB1	>2000	<9.15
		DON	>2000	<9.15
DON	170	AFB1	>2000	<8.50
		ZEN	>2000	<8.50

**Table 3:** Comparison of the detected AFB1, ZEN and DON concentrations obtained by this method and its certified concentrations in CRMs.

Target Analyte	Grain Sample	Certified Value (μg/kg)	Detected (μg/kg)	Bias%
AFB1	Corn	28	23.1 ± 0.83	−17
		7.4	6.92 ± 0.60	−6
		2.2	2.26 ± 0.80	2
	Rice	14.3	15.2 ± 0.56	3
		8.7	8.96 ± 0.33	8
		2.6	2.27 ± 0.20	−12
ZEN	Corn	85	84.0 ± 3.71	−2
		750	803 ± 19.2	7
	Rice	32	29.1 ± 3.47	−9
		55	52.3 ± 10.1	−5
		380	335 ± 12.9	−11
DON	Corn	1310	1510 ± 120	15
		400	419 ± 7.82	5
		125	119 ± 12	−6
	Rice	536	571 ± 18.8	7
		720	737 ± 17.4	3

## Data Availability

All data are contained within the article.

## Supplementary Materials

The following supporting information can be downloaded at: https://www.mdpi.com/article/10.3390/molecules28073260/s1. Figure S1: Optimization of the coating concentration on T lines. (a) The inhibition rate of spiked samples with different concentrations of AFB1-BSA; (b) T line fluorescence intensity of different concentrations of AFB1-BSA. (c) The inhibition rate of spiked samples with different concentrations of ZEN-BSA; (d) T line fluorescence intensity of different concentrations of ZEN-BSA. (e) The inhibition rate of spiked samples with different concentrations of DON-BSA; (f) T line fluorescence intensity of different concentrations of DON-BSA.
