# Supplementary material for: “Green” Extraction and On-Site Rapid Detection of Aflatoxin B1, Zearalenone and Deoxynivalenol in Corn, Rice and Peanut
Source: Molecules. 2023 Apr 6;28(7):3260. doi: 10.3390/molecules28073260 (PMC10096640; doi:10.3390/molecules28073260)
Supplement: Supplementary file 1 [file molecules-28-03260-s001.zip › molecules-2294737-supplementary.pdf]

"Green" Extraction and On-Site Rapid Detection of Aflatoxin B1, Zearalenone and Deoxynivalenol in Corn, Rice and Peanut

Zijing Li, Zepeng Li, Xintong Li, Qi Fan, Yinuo Chen and Guoqing Shi*

School of Chemistry and Biological Engineering, University of Science and Technology Beijing, Beijing, 100083, China; lizijing09@163.com (Z.L.); 13021230300@163.com (Z.L.);
m202110884@xs.ustb.edu.cn (X.L.); qi.fan17@outlook.com (Q.F.);
18612203588@163.com (Y.C.)

* Correspondence: shiguoqing@ustb.edu.cn; Tel.: +86-10-62334497

1. Optimization of preparation conditions of TRF-LFIA test strips

According to different mycotoxins, different concentrations of antigen were used to investigate the change in T line fluorescence intensity and inhibition rate. First, the coating concentrations of AFB1-BSA on the T line were set as 0.1, 0.2, 0.3, 0.4 and 0.5 mg/ml. The blank samples and the spiked samples (10 μg/kg) were detected at the same time. When the T line concentration was 0.3, 0.4 and 0.5 mg/ml (Fig. S1a), the T line fluorescence intensity of blank samples and spiked samples did not obviously change, and there was no significant difference. Figure S1b shows that the inhibition rate reached the highest and tended to be stable when the T line concentration was 0.3 mg/ml. Therefore, AFB1-BSA concentration of 0.3 mg/ml was selected as the optimal concentration. Next, the coating concentrations of ZEN-BSA were set as 0.01, 0.03, 0.05, 0.07 and 0.1 mg/ml. Then, the blank samples and the spiked 300 μg/kg DON samples were detected simultaneously. With the increase in the T line concentration, the fluorescence intensity of the T line increased and reached the highest value after 0.07 mg/ml, as shown in Fig. S1c and S1d. When the T line concentration was increased to 0.1 mg/ml, it did not increase significantly, and when the inhibition rate was 0.05 mg/ml, it tended to be stable and did not change significantly. Therefore, the T line concentration of 0.05 mg/ml was selected as the optimal concentration of ZEN-BSA. Finally, the coating concentrations of DON-BSA were set as 0.1, 0.3, 0.5, 0.6, and 0.7 mg/ml. As shown in Fig. S1e and S1f, the T-line fluorescence intensity of blank samples and spiked 100 μg/kg samples did not change evidently when the T line concentrations

were 0.5, 0.6, and 0.7 mg/ml. Similarly, the inhibition rate of spiked samples did not change considerably at different concentrations, and there was no significant difference. Therefore, the T line concentration of 0.5 mg/ml was selected as the optimal concentration of DON-BSA.

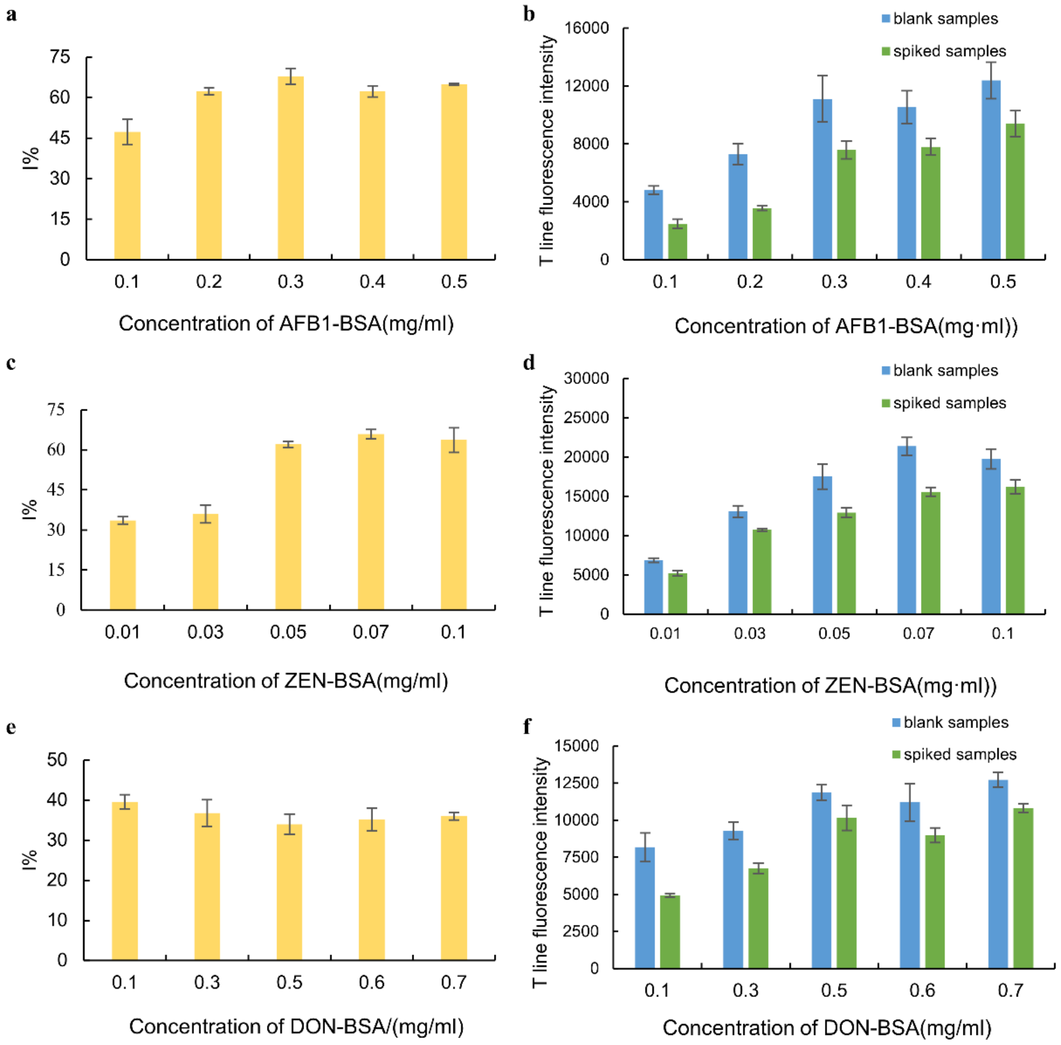

Figure.S1 Optimization of the coating concentration on T lines. (a) The inhibition rate of spiked samples with different concentrations of AFB1-BSA; (b) T line fluorescence intensity of different concentrations of AFB1-BSA. (c) The inhibition rate of spiked samples with different concentrations of ZEN-BSA; (d) T line fluorescence intensity of different concentrations of ZEN-BSA. (e) The inhibition rate of spiked samples with

different concentrations of DON-BSA; (f) T line fluorescence intensity of different concentrations of DON-BSA.
